# Different Patterns of Sleep-Dependent Procedural Memory Consolidation in Vipassana Meditation Practitioners and Non-meditating Controls

**DOI:** 10.3389/fpsyg.2019.03014

**Published:** 2020-01-23

**Authors:** Elizaveta Solomonova, Simon Dubé, Cloé Blanchette-Carrière, Dasha A. Sandra, Arnaud Samson-Richer, Michelle Carr, Tyna Paquette, Tore Nielsen

**Affiliations:** ^1^Dream and Nightmare Laboratory, Centre for Advanced Research in Sleep Medicine, CIUSSS NÎM – HSCM, Montréal, QC, Canada; ^2^Department of Psychiatry, Université de Montréal, Montréal, QC, Canada; ^3^Culture, Mind and Brain Research Group, Department of Psychiatry, McGill University, Montréal, QC, Canada; ^4^Department of Psychology, Concordia University, Montréal, QC, Canada; ^5^Integrated Program in Neuroscience, McGill University, Montréal, QC, Canada; ^6^Sleep Laboratory, Swansea University, Swansea, United Kingdom

**Keywords:** procedural memory, memory consolidation, vipassana meditation, REM sleep, NREM sleep, sleep spindles, body awareness

## Abstract

**Aim:**

Rapid eye movement (REM) sleep, non-rapid eye movement (NREM) sleep, and sleep spindles are all implicated in the consolidation of procedural memories. Relative contributions of sleep stages and sleep spindles were previously shown to depend on individual differences in task processing. However, no studies to our knowledge have focused on individual differences in experience with Vipassana meditation as related to sleep. Vipassana meditation is a form of mental training that enhances proprioceptive and somatic awareness and alters attentional style. The goal of this study was to examine a potential role for Vipassana meditation experience in sleep-dependent procedural memory consolidation.

**Methods:**

Groups of Vipassana meditation practitioners (*N* = 22) and matched meditation-naïve controls (*N* = 20) slept for a daytime nap in the laboratory. Before and after the nap they completed a procedural task on the Wii Fit balance platform.

**Results:**

Meditators performed slightly better on the task before the nap, but the two groups improved similarly after sleep. The groups showed different patterns of sleep-dependent procedural memory consolidation: in meditators, task learning was positively correlated with density of slow occipital spindles, while in controls task improvement was positively associated with time in REM sleep. Sleep efficiency and sleep architecture did not differ between groups. Meditation practitioners, however, had a lower density of occipital slow sleep spindles than controls.

**Conclusion:**

Results suggest that neuroplastic changes associated with meditation practice may alter overall sleep microarchitecture and reorganize sleep-dependent patterns of memory consolidation. The lower density of occipital spindles in meditators may mean that meditation practice compensates for some of the memory functions of sleep.

## Introduction

A wealth of research supports a role for sleep in explicit and implicit memory consolidation (for reviews see Diekelmann and Born, 2010; Stickgold, 2013; Shönauer and Gais, 2017). Sleep is thought to strengthen information learned during the day, to select which experiences are best remembered and which are best forgotten, and to assimilate new knowledge into existing autobiographical networks (Walker and Stickgold, 2010; Rolls et al., 2013; Stickgold and Walker, 2013; Deliens and Peigneux, 2014). While most research to date has focused on the effects of a full night of sleep, an increasing number of studies report that daytime naps have effects that are similar to those of nighttime sleep on memory processes (Mednick et al., 2003; Backhaus and Junghanns, 2006; Nishida and Walker, 2007; Lahl et al., 2008; Seeck-Hirschner et al., 2010; Fogel et al., 2014; Lo et al., 2014; Korman et al., 2015).

Current research in cognitive science focuses strongly on contemplative practices and their effects on physical and mental health. Such practices include an element of cultivating access to and awareness of increasingly subtle bodily sensations (Kerr et al., 2013; Zeng et al., 2014). Meditation practitioners can be considered an “expert” group of healthy individuals who intentionally cultivate attention to bodily states and are better able to access and employ them (Fox et al., 2012), thus potentially embodying some cognitive processes differently. However, it is still unknown whether meditators may rely more upon awareness of somatosensory and other types of endogenous information to encode memories for skill learning.

In this study, we investigate whether Vipassana, a specific meditation practice focusing on body awareness, changes the neurobiological qualities of sleep-dependent memory consolidation. Specifically, we chose a full body procedural memory task and a daytime nap protocol to test whether: (1) meditation practitioners and meditation-naïve controls differ in performance and improvement on a procedural balance task; (2) the two groups differ in their patterns of sleep stage and sleep microarchitecture in reaction to the task; and (3) daytime nap sleep characteristics (sleep stages and sleep spindles) in meditation practitioners are similar in quality to nighttime sleep.

Consolidation of procedural memory – skill learning – during sleep has been studied using a number of tasks, including sequential finger tapping (Benedict et al., 2009; Doyon et al., 2009; Van Der Werf et al., 2009; Dresler et al., 2010; Genzel et al., 2011; Holz et al., 2012b; Wamsley et al., 2012; Antonenko et al., 2013), serial-reaction time task (Galea et al., 2010; Prehn-Kristensen et al., 2011; Ertelt et al., 2012), motor sequence task (Tucker and Fishbein, 2009; Manoach et al., 2010), mirror-tracing task (Smith et al., 2004b; Javadi et al., 2011; Puetz et al., 2011; Holz et al., 2012a), button-box sequence (Wilhelm et al., 2012), visuomotor adaptation task (Doyon et al., 2009); texture discrimination task (Gais et al., 2008; Cipolli et al., 2009), visual discrimination task (Suzuki et al., 2012), and others. These tasks usually involve fine motor skills using the fingers or hand-eye coordination, but do not typically involve balance or bodily displacements. Tasks involving balance skills or full-body learning have only rarely been used in memory consolidation research (Buchegger and Meier-Koll, 1988; Buchegger et al., 1991; Tjernstrom et al., 2005). While hand-eye coordination tasks are well-validated and are easy to implement, full-body tasks may be more representative and ecologically valid with relation to global procedural memory skills. Many daily behaviors considered as procedural skills rely on greater involvement of vestibular and kinesthetic systems (e.g., navigating different spaces, sports, bicycling, cooking, tool use, etc.), and some involve fine motor skills (e.g., typing, using smartphones, specialized tool use). Thus, full-body procedural learning may provide additional insights into global processes underlying sleep-dependent memory consolidation.

Some studies suggest particular sleep macro (sleep stages) and micro architecture aspects to be important for procedural memory consolidation. For example, NREM sleep duration (Walker et al., 2002) as well as NREM electrophysiological events, such as sleep spindles (Barakat et al., 2011; Fogel et al., 2017), have been associated with post-sleep task improvement. The sleep spindles, phasic events (0.5- to 3.0-s duration) characterized by bursts of 11–16 Hz EEG activity occurring predominantly during stage 2 NREM (N2) sleep, are of particular interest. Converging evidence points to possible different neural mechanisms implicated in fast and slow sleep spindles (Molle et al., 2011), but few studies to date have examined the combined contribution of slow and fast sleep spindles to memory consolidation. In one recent study, fast (but not slow) sleep spindles were involved in transfer or implicit knowledge to more explicit awareness (Yordanova et al., 2017). In another study, consolidation of face memory, which can be considered as having both explicit and implicit components, was associated with both fast and slow sleep spindles (Solomonova et al., 2017).

Sleep’s role in memory consolidation is not only influenced by task type and sleep stage/sleep microarchitecture, but also by individual differences in learning abilities and cognitive styles. In one study, for example the ability to bring a motor task into explicit awareness improved post-sleep performance on the task (Robertson et al., 2004). This suggests that training in a particular kind of attentional engagement with the learning task may change the neurocognitive style of offline memory processing and affect performance. Contemplative practices (e.g., meditation and mindfulness) can be considered as one particular way of attentional engagement. Vipassana meditation is one such practice. It is characterized by developing sustained and systematic practice of being aware of one’s bodily sensations, with an aspiration to ultimately gain insight into the nature of the mind (Hart, 1987; Goenka, 1997; Chavan, 2008). Practitioners typically start with a focused attention practice – mindfulness of breathing – and continue with the practice of the body scanning technique, which is the main particularity of Vipassana. During the second part of the practice, meditators are instructed to mentally examine their bodily states to become aware of subtle sensations and, ultimately, to approach them with equanimity. This contemplative tradition may change practitioners’ cognitive style in a global way, including changing processes of attention, memory encoding, consolidation and retrieval.

Studies show that Vipassana meditation yields beneficial effects of decreasing stress and improving well-being (Szekeres and Wertheim, 2015), helping generate greater perceptual clarity and decrease automated reactivity to stimuli (Cahn and Polich, 2009; Cahn et al., 2013; Delgado-Pastor et al., 2013), and decreasing anger, hostility and depressive symptoms (Kasai et al., 2015). Other studies report improved psychological well-being (Montero-Marin et al., 2016) and cognitive flexibility (Kasai et al., 2015). Other effects of Vipassana training include increased somatosensory awareness, e.g., increased awareness of pain, more spontaneous body movements, increased mindfulness, and development of equanimity, or the ability to adapt to extreme changes in lived experience (Kornfield, 1979). Sleep architecture changes of Vipassana practitioners are few, but include longer REM sleep periods when compared to controls and yoga practitioners (Sulekha et al., 2006). Longer-term practitioners (over 7 years of daily practice) have more N3 sleep, less N2 sleep, fewer awakenings from sleep and an altered pattern of REM sleep microarchitecture (Maruthai et al., 2016).

Although it may seem intuitive to consider that meditation practice affects learning and memory by enhancing specific cognitive and attentional skills (Valentine and Sweet, 1999; Jha et al., 2007; Zeidan et al., 2010), few studies to date have directly addressed the effect of meditation on memory consolidation, with mixed results. One study reports very few differences between long-term meditation practitioners (including Vipassana meditators) and non-meditating controls: meditators showed better performance on short-term and free recall long-term memory (Lykins et al., 2012). Other studies report improvements in working memory in meditation practitioners (Mrazek et al., 2013; Basso et al., 2019), specifically, in a military cohort (Jha et al., 2010), and in a group of adolescents (Quach et al., 2016). With respect to procedural motor memory, only one other study to our knowledge assessed contribution of post-training meditation practice to memory consolidation (Immink, 2016): experienced yoga *nidra* meditation practitioners showed post-training memory benefits on a sequence tapping task.

In sum, both sleep stages and sleep microarchitecture have been associated with procedural learning. The patterns of sleep-dependent learning may reflect individual learning styles and thus may be different in meditation practitioners. Vipassana meditation may produce changes in body awareness, which in turn may influence not only individual health and cognitive patterns, but also the style of encoding of procedural memory. In the current study, we hypothesize that: (1) following a daytime nap, Vipassana meditators (MED group) will show more improvement on a procedural task than will meditation-naïve controls (CTL group). We also hypothesize (2) that the MED and CTL groups will express two distinct neurobiological learning patterns: the MED group will show a NREM-dependent pattern, reflecting a more explicit learning process; more specifically, fast sleep spindles will correlate with task improvement. In contrast, the CTL group will show a REM-dependent pattern, consistent with previous research on non-declarative learning.

## Materials and Methods

### Participants

Forty-two male (*n* = 21) and female (*n* = 21) participants between the ages of 18 and 35 years (*M* = 25.4 ± 4.4) were recruited for a daytime nap study via online advertisements, and subsequently screened via phone or online questionnaire. In the MED group there were 22 participants (*M*age = 25.8 ± 4.1, 11 men, 11 women) and in the CTL there were 20 participants (*M*age = 25.0 ± 4.8, 10 men, 10 women). Inclusion criteria for both groups were: 18–35 years of age, high dream recall (3+ per week), good self-reported physical/mental health, no sleep disturbances (e.g., shift work or jet lag). To be included in the MED group, Vipassana practitioners had to have taken part in at least one 10-day retreat, which consists of approximately 100 h of practice, and to be currently practicing Vipassana meditation on at least a weekly basis. Participants completed an informed consent form approved by the Hôpital du Sacré-Coeur de Montréal ethics board. They were financially compensated for the time spent in the laboratory, parking or public transit, and lunch expenses.

### Body Awareness

Body awareness was measured using the Scale of Body Connection (SBC) questionnaire (Price and Thompson, 2007) This scale consists of 20 items each scored on 0–4 scales and which produces two independent subscales: body awareness (12 items) with items that assess awareness of, e.g., tension, bodily stress, breath, and emotional state, and bodily dissociation (eight items) with items that assess, e.g., feeling frozen/numb, separated from the body, and difficulty in expressing emotions. Subscale scores consist of the average ratings of constituent items (0–4). The questionnaire has been validated on studies of bodily awareness in women with substance abuse disorder (Price et al., 2012), of exercise and body awareness therapy in major depression (Danielsson et al., 2014), of body therapy for survivors of childhood sexual abuse (Price, 2007), and of interoceptive awareness of breathing in experienced meditation practitioners (Daubenmier et al., 2013).

### Procedure

Participants arrived in the laboratory at 9:00 am and completed a consent form and a battery of questionnaires, which included the SBC. They then completed the procedural task and had the PSG setup installed. Immediately before lights-out, the MED group meditated in bed for 10 min and the CTL group simply relaxed for the same amount of time. All participants were then given a window of opportunity to sleep of approximately 90 min. They were awakened twice with a non-stressful, 80 dB, 500 Hz, once at sleep onset and once at the end of the nap. At these times, spontaneous dream reports and dream questionnaires were completed. Following the second awakening, electrodes were removed and participants repeated the procedural task.

### Procedural Memory (Balance) Task

Participants performed a procedural memory task requiring whole body balance, i.e., a video game entitled “Balance bubble” for the Nintendo Wii Fit Balance Board. In this task, participants control a virtual character that moves along a river in a bubble by shifting their weight on the Balance Board. The objective is to complete the river path as quickly as possible without touching the river’s edges and thus bursting the bubble. Bursting the bubble required the participant to start again from the beginning of the path. The task was performed on a 42-in. television screen. All participants were assessed for any vision correction and instructed to wear their glasses or contact lenses if needed. Participants had a 2-min period to acquaint themselves with the task, and to ask questions of the experimenter, who ensured that participants clearly understood the requirements of the game. The total maximum game time was 90 s, and participants were allowed to repeat the task until a total of 5 min of gameplay had elapsed. Participants were instructed to attempt to complete the task as many times as possible during the 5-min period. The task was performed once before lights-out (T1) and once after awakening (T2).

The following variables were calculated to assess performance on the task: highest score on all attempts averaged; the number of times participants completed the task by arriving at the end of the “river”; average score in game “meters” over all attempts at the game, and average time spent correctly balanced in the bubble over all attempts. Two measures of improvement on the task were used: T2–T1GameScore (average score after nap minus average score before nap), and T2–T1Time (average time after nap minus average time before nap).

### Polysomnography

Participants slept in a quiet bedroom under continuous audio-visual monitoring with a two-way intercom. A standard polysomnographic montage was used: six EEG channels (F3, F4, C3, C4, O1, O2) referenced to A1 and re-referenced to A1 + A2 offline; four electrooculography (EOG) channels (horizontal and vertical), and three electromyography (EMG) channels (chin, wrist, leg). Acquisition of EEG signals was done using an M15 Grass Acquisition System (−6 dB filters with cut-offs at 0.30 and 100 Hz) and Harmonie v6.2b software (Natus Medical Incorporated, Pleasanton, CA, United States). Sleep stages were scored according to standard criteria (Iber, 2007) by an experienced technician and standard sleep variables were calculated using in-house software.

Sleep spindles were detected on artifact-free sleep epochs recorded from F3, F4, C3, C4, O1, and O2 derivations by an in-house detector. The full detection algorithm is described by Nielsen et al. (2016) and by O’Reilly and Nielsen (2014). Spindles were separated into slow (10.00–12.99 Hz) and fast (13.00–16.00 Hz) types and densities calculated as the number of spindles of each type divided by time elapsed in N2 sleep.

## Results

### Body Awareness

A total of 32 participants (15 MED and 17 CTL; 80%) returned questionnaires by mail. Independent samples *t*-tests on the SBC score and Body Awareness and Bodily Dissociation subscales revealed, as predicted, that the MED group scored significantly higher on Body Awareness than did the CTL group (M(MED) = 0.243 ± 0.060; M(CTL) = 0.194 ± 0.061; *t*(30) = −2.289; *p* = 0.029; Cohen’s *d* = −0.81; 95% CIs [−1.529, −0.081]) but did not differ on Bodily Dissociation. Body Awareness was not correlated with years of meditation experience in the MED group (*r* = −0.471, *p* = 0.077).

The MED group showed positive correlations between Body Awareness and average time (*r* = 0.580; *p* < 0.05) and score (*r* = 0.518; *p* < 0.05). In contrast, CTL participants showed no significant correlations were observed between Body Awareness scores and task performance measures.

### Task Performance

Separate repeated-measures ANOVAs with average time spent balanced, average score, and highest score obtained as within subjects factors and with condition (MED or CTL) as between subject factors revealed that both groups improved on average time (*F*(1) = 20.172, *p* < 0.001), score (*F*(1) = 60.564, *p* < 0.001), and highest score obtained (*F*(1) = 29.546, *p* < 0.001). No group effect was observed for either time (*F*(1) = 0.209, *p* = 0.650) or score (*F*(1) = 0.477, *p* = 0.494). A significant group effect, however, was observed for highest score obtained (*F*(1) = 4.474, *p* = 0.041). The follow-up independent samples *t*-test showed that the highest score obtained at T1 was significantly higher in MED than in CTL (M(MED) = 1028.4 ± 142.21; M(CTL) = 913.3 ± 187.54; *t*(38) = 2.188; *p* = 0.035).

In addition, meditators finished the game more often, especially after the nap: 2 (10%) of CTL participants finished the task at least once before the nap (T1), compared with 5 (25%) of MED participants (χ2 = 0.693, *p* = 0.405, 2-tailed, Yates corrected) whereas 6 (30%) of CTL participants finished the task after the nap (T2) compared with 12 (60%) of MED participants (χ2 = 3.636, *p* = 0.057). No other differences in task scores were observed between the groups.

### Sleep Structure

Two MED participants was dropped from spindle analyses due to insufficient sleep duration. No statistically significant differences between MED and CTL groups were observed for any sleep characteristic, except for a trend (*p* = 0.09) for CTL participants toward overall longer sleep duration (see Table 1 for complete results).

**TABLE 1:** Sleep measures for Vipassana meditators (MED: *N* = 20) and non-meditating controls (CTL: *N* = 20).

Sleep characteristic	Mean MED ± SD	Mean CTL ± SD	*t*	df	*p*	Cohen’s *d*
Total sleep duration (min)	65.98 ± 25.27	78.30 ± 19.32	–1.73	38.00	0.09^†^	0.55
Sleep latency (min)	8.78 ± 6.75	14.35 ± 20.08	0.20	38.00	0.84	–0.06
REM latency (min)	16.35 ± 22.35	20.74 ± 31.23	–0.47	32.00	0.64	0.16
Sleep efficiency	73.66 ± 23.10	81.70 ± 13.31	–1.31	29.68	0.20	0.42
N1 duration (min)	7.13 ± 7.67	7.03 ± 3.81	0.05	38.00	0.96	–0.02
N2 duration (min)	32.83 ± 16.47	36.05 ± 15.05	–0.65	38.00	0.52	0.20
N3 duration (min)	15.05 ± 13.38	22.53 ± 18.99	–1.44	38.00	0.16	0.46
Total NREM duration (min)	55.00 ± 23.28	65.60 ± 22.37	–1.47	38.00	0.15	0.46
REM duration (min)	10.98 ± 6.12	12.70 ± 9.99	–0.66	38.00	0.51	0.21
Wake duration (min)	24.75 ± 24.71	17.45 ± 12.33	1.18	27.91	0.24	–0.37
N1%	13.31 ± 16.30	10.45 ± 8.94	0.69	38.00	0.50	–0.22
N2%	50.55 ± 17.18	45.10 ± 15.68	–0.90	38.00	0.50	–0.29
N3%	19.46 ± 15.95	26.45 ± 20.43	–1.21	38.00	0.24	0.38
NREM%	83.32 ± 10.68	82.90 ± 15.17	0.10	38.00	0.92	–0.03
REM%	16.69 ± 10.68	17.11 ± 15.17	–0.10	38.00	0.92	0.03
Wake%	26.29 ± 24.02	18.30 ± 13.31	1.30	29.66	0.20	–0.41
*N* awakenings	5.65 ± 3.84	6.70 ± 4.18	–0.83	38.00	0.41	0.26

*^†^*p* < 0.10.*

### Relationship Between Sleep and Meditation Experience

To test the relationship between cumulative lifetime meditation experience and sleep architecture, Spearman correlations analyses were performed for self-reported meditation experience in the MED group (in hours), and duration of individuals sleep stages (N1, N2, N3, REM, in minutes). We observed a strong negative correlation between cumulative lifetime meditation experience and time in N2 sleep (*r*_*s*_ = −0.546, *p* = 0.015) but no correlations with other sleep stages.

### Sleep Spindles

**FIGURE 1:**
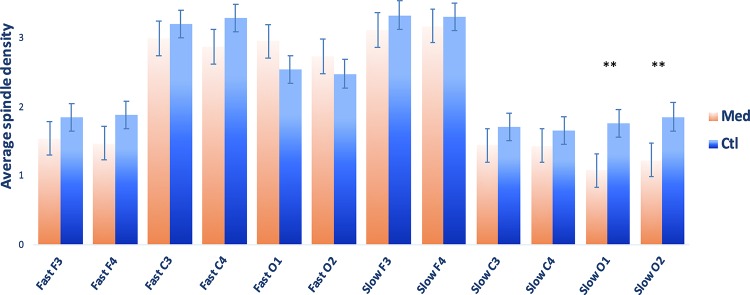
Sleep spindle densities (M ± SEM) for Vipassana meditators (MED; *N* = 20) and non-meditating controls (CTL; *N* = 20). Spindle density calculated as the number of sleep spindles/time in NREM2 sleep. ^∗∗^*p* < 0.05.

A total of 20 CTL and 19 MED subjects were compared (Figure 1 for group comparisons of spindle density). Distribution of spindle density was moderately to highly skewed for most spindle measures, thus non-parametric Mann–Whitney independent samples *U*-tests were used for analyses involving spindle measures. There were no significant group differences in total spindle density or fast spindle density. However, the MED group showed lower slow spindle densities in occipital derivations, specifically: a lower spindle density in O1 (M(MED) = 1.077 ± 0.819; M(CTL) = 1.754 ± 0.995; *U* = 277; *p* = 0.015; *r* = 0.461; 95%CIs [0.130, 0.699]) and a lower slow spindle density in O2 (M(MED) = 1.225 ± 0.842; M(CTL) = 1.848 ± 1.035; *U* = 264; *p* = 0.039; *r* = 0.389; 95%CIs [0.044, 0.652]). See Figure 1 for complete spindle comparison between groups.

### Sleep and Procedural Memory Consolidation

To assess the relationships between sleep and improvement on the balance task, the two most sensitive task measures were used: change in average *time* spent in balance (T2–T1) and change in average *score* (T2–T1). The decision to use these scores was based on the fact that *time* and *score* represented two main facets of successful performance on the task. Time spent in balance (even when score was low) showed how stable participants were on the platform, and score one the task (independent of how fast the task was completed) showed how far they got in the game. Spearman correlations were calculated to evaluate dose-response relationships between change in sleep stages, task performance, and slow and fast spindle density.

### Sleep Stages

For the MED group, there were no statistically significant relationships between task improvement scores (average time and average score) and sleep stages (NREM: N1, N2, N3, and REM). For CTL group, improvement on average time spent balanced correlated negatively with N1 duration (*r* = −0.465, *p* = 0.007) and positively with REM sleep duration (*r* = 0.592, *p* = 0.006). Further, improvement on average score correlated negatively with N1 duration (*r* = −0.470, *p* = 0.037); and positively with REM sleep duration (*r* = 0.536, *p* = 0.015). Improvement on average score also correlated with several other sleep measures which were not predicted, i.e., negatively with sleep latency (*r* = −0.475, *p* = 0.034); positively with sleep efficacy (*r* = 0.451, *p* = 0.046); and negatively with wake duration (*r* = −0.498, *p* = 0.025). See Table 2 for complete results.

**TABLE 2:** Pearson correlation coefficients between sleep characteristics and post-nap improvement in performance on a procedural task in meditators (MED) and controls (CTL).

	MED		CTL			
Sleep variable	Time	Score	Time		Score	
Total sleep duration	0.31	0.06	–0.10		0.01	
Sleep latency	0.16	0.20	–0.20		–0.48	**
REM latency	–0.02	0.18	0.28		0.26	
Sleep efficiency	0.20	0.04	0.29		0.45	**
N1 duration	0.01	–0.09	–0.47	**	–0.47	**
N2 duration	0.22	0.14	–0.39	*	–0.23	
N3 duration*	0.20	–0.01	–0.01		0.01	
Total NREM duration*	0.30	0.06	–0.35		–0.23	
REM duration	0.11	0.00	0.59	***	0.54	**
Wake duration	–0.15	–0.02	–0.32		–0.50	**
N1%	–0.11	–0.14	–0.41	*	–0.42	^∗^
N2%	0.02	0.20	–0.42	*	–0.33	
N3%	0.12	–0.07	–0.01		0.00	
NREM%	0.05	–0.01	–0.66	***	–0.57	***
REM%	–0.05	0.01	0.66	***	0.57	***
Wake%	–0.20	–0.04	–0.29		–0.45	**

Average improvement in time and in score is reported. *p < 0.05; **p < 0.01; ***p < 0.001 (Conservative error rate adjustment for 32 correlations per group = 32/0.05 = 0.002).

### Sleep Spindles

Spearman correlations between measures of task improvement (T2–T1Time and T2–T1GameScore) and sleep spindle densities revealed significant relationships for the MED group, but not the CTL group. T2–T1Time correlated positively with density of slow sleep spindles in O1 (*r_s_* = 0.463, *p* = 0.047). T2–T1GameScore correlated positively with density of slow sleep spindles in O1 (*r_s_* = 0.489, *p* = 0.035); and showed negative trend correlations with density of fast sleep spindles in O1 (*r_s_* = −0.400, *p* = 0.091); and negatively with density of fast sleep spindles in O2 (*r_s_* = −0.391, *p* = 0.099).

## Discussion

This study is the first to examine sleep-dependent memory consolidation in meditation practitioners. Although meditators were found to perform slightly better on the balance task at first testing than controls (meditators were able to complete the task more often), both groups improved to a similar extent after a morning nap. However, distinct patterns of relationships between task improvement and sleep structure were observed for the two groups: meditators showed a relationship between task improvement and occipital NREM sleep spindles while controls showed a relationship between task improvement and REM sleep time. In addition, we found differences in spindle density between meditators and controls, with meditators having significantly lower spindle density in occipital regions. These results together suggest that meditation experience may contribute to large-scale changes in the electrophysiological indicators of neuroplasticity during sleep.

### Different Sleep-Dependent Memory Consolidation Styles in Meditators and Controls

Results support the study’s main hypothesis that Vipassana meditation practitioners, potentially by virtue of training in attending to bodily states and stimuli, may rely upon a different neurobiological learning style, one that manifests in distinct changes in sleep microarchitecture. Specifically, meditation practitioners showed relationships between the density of slow and fast occipital spindles in N2 sleep and task improvement. While slow occipital spindle density correlated positively with task improvement, fast occipital spindle density showed a trend toward the opposite. Meditators did not, however, show any links between duration of REM or NREM sleep stages and task improvement. On the other hand, non-meditating controls did show a strong positive correlation between time in REM sleep and task improvement. This is consistent with previous research where, in a series of studies, a novel motor skill, in this case jumping on a trampoline, was associated with increased REM sleep (Buchegger and Meier-Koll, 1988; Buchegger et al., 1991). Our results partially replicate these findings to the extent that improvement on our procedural balance task was associated with length of REM sleep during the daytime nap for control participants only. They did not, however, show any links between sleep spindles, especially fast sleep spindles, and task improvement. Both of these findings have been reported in previous studies. To illustrate, for the N2 spindle finding among meditators, we previously reported similarly perpendicular relationships between fast and slow spindles in relation to learning: fast N2 spindles correlated positively and slow spindles correlated negatively with face recognition (Solomonova et al., 2017). For the REM sleep finding in controls, earlier research similarly linked duration of REM sleep with implicit memory consolidation (Karni et al., 1994). We also report a relationship between performance on the balance task and other sleep variables for the non-meditating control group. For instance, moderate correlations were found between overall sleep quality and improvement on task (positive correlation with sleep efficacy and negative correlations with wake duration and with N1 duration). This potentially reflects the overall beneficial role for sleep quality (higher efficiency, less fragmentation) in memory consolidation. In addition, higher sleep efficacy, lower N1 sleep time and lower wake time are all related to higher chances of reaching REM sleep during the nap, and thus are likely related to our REM sleep finding.

### Different Styles of Post-Task Sleep-Dependent Processing?

One interpretation of these findings is that meditation practitioners approach a procedural full-body balance task in a more “explicit” manner, in the sense that they are able to bring to awareness elements of their somatic and bodily experience that usually remain unconscious among untrained individuals. Their greater familiarity with bodily sensations may thus have allowed them to perform somewhat better on some, but not all, aspects of the task. They performed slightly better than the control group on the task overall, achieving higher maximum scores both before and after the nap. This advantage may reflect a shift in the neuroplasticity mechanisms that were brought to bear on the task during sleep. This idea is consistent with the suggestion (Smith et al., 2004a) that REM sleep is associated with processing of complex tasks while Stage 2 sleep benefits the consolidation of simpler tasks. Smith et al. (2004a) propose that the involvement of REM or Stage 2 sleep in consolidation of a motor task depends on perceived complexity of the task; participants who are less skillful at the outset will show a dependence on REM sleep and those who find the task easier will show a N2 dependence. Because our meditators scored higher in body awareness and possibly found the task slightly easier, they may have needed only N2 spindle mechanisms to refine their learning. In contrast, if control participants found the task more complex or novel, they may have had a greater need for REM sleep control mechanisms.

Despite the feasibility of this explanation, the significance of several additional findings from the current study remain unclear. One is the question of why meditators had a lower rate of occipital spindles when sleep spindle density was associated with task improvement. A second outstanding question is why slow rather than fast sleep spindle densities were associated with task improvement. Many previous studies (Tamaki et al., 2009; Barakat et al., 2011; Lustenberger et al., 2016) report that fast spindle density is preferentially correlated with improvement on procedural tasks. However, a growing body of evidence supports the notion that slow spindles are also a reliable correlate of procedural learning in some contexts. Nishida et al. (2016) showed a negative association between slow spindle power and a finger-tapping motor tracing task in depressed but not in healthy participants. A second group (Holz et al., 2012a) showed a positive correlation between slow spindle activity (sigma: 12–14 Hz) in NREM sleep and improvement on a mirror tracing task. Third, studies have shown that slow spindles are associated with explicit memory consolidation on tasks such as word-lists (Holz et al., 2012a) and auditory verbal learning and declarative learning in epileptic patients (Del Felice et al., 2015). Fourth, in children, slow sleep spindle activity was found to be associated with declarative learning efficiency (Hoedlmoser et al., 2014). Finally, in a study using daytime naps (Schmidt et al., 2006), participants learning difficult and easy word-pairs showed evidence of slow spindle associations with improvement only on the difficult pairs. Such converging evidence links slow spindles to procedural learning and is consistent with our finding that meditators reacted to the balance task as a procedural task requiring N2 sleep mediation.

### Possible Napping Profile

A second possible explanation of our group differences in sleep-dependent effects is that meditators are conforming to a profile known to characterize habitual nappers, either because they are used to taking frequent naps or because periods of daytime meditation may confer advantages similar to those provided by sleep, especially theta-rich Stage 1 NREM sleep (Aftanas and Golosheykin, 2005). Unfortunately, our findings do not further clarify the first point because we did not collect information about habitual napping practices. Yet, there is evidence (Milner et al., 2006) that individuals habitually taking daytime naps show a relationship between motor task improvement and sleep spindles whereas non-habitual nappers do not. Such findings, together with evidence that meditation practitioners commonly fall asleep during meditation sessions (Pagano et al., 1976), provides some support for the notion that meditation confers either sleep-dependent or sleep-like advantages in memory consolidation. However, more research taking into account the habitual napping practices of participants is clearly needed for this explanation to be considered probable.

The notion that meditation practice leads to a different style of learning is consistent with both the philosophy of meditation approaches and a growing body of research. Indeed, the underlying goal of most traditional meditation practices, including Vipassana meditation, is not simple training of attentional acuity, but rather development of insight into one’s own patterns of reactivity in order to decrease unwholesome traits and increase wholesome traits and behaviors (Hart, 1987; Goenka, 1997). Ultimately, the goal is not to improve at meditation, but to improve at life skills more generally. Thus, neuroplastic changes and acquired mental skills associated with meditation practices may have an important effect on changing one’s cognitive, emotional and memory patterns more broadly (Hasenkamp and Barsalou, 2012). This idea is consistent with previous findings, including those from a study wherein mindfulness training led to increased somatosensory awareness of the experience of sadness, which in turn was related to decreased depression (Farb et al., 2010). Neurophysiologically, meditation training may change the functional connectivity associated with bodily representation and thereby recruit the interoceptive skills that underlie several perceptual and cognitive tasks (Farb et al., 2013). Our findings are thus consistent with the notion that Vipassana practice, through increased interoception and body awareness, facilitates the development of cognitive skills and thereby differentially influences sleep’s reactions to new procedural learning tasks.

### Task Performance, Body Awareness, and Bodily Dissociation

We previously reported (Solomonova, 2018), that meditation practitioners did not differ from non-meditating controls on most aspects of task performance. As predicted, however, meditators scored higher than controls on the Body Awareness subscale of the Scale of Body Connection and these scores were related to task performance (time and score) before the nap. These finding are consistent with studies suggesting that sustained meditation practice contributes to higher levels of somatic awareness (Fox et al., 2012) and confirms our rationale for choosing a full-body balance task to examine patterns of sleep-dependent memory consolidation. In this case, one required skill for learning the task quickly is a heightened level of attunement to one’s own bodily/somatic states.

### Sleep and Spindle Differences Between Meditators and Controls

#### Sleep Architecture

In contrast to existing literature on sleep in Vipassana practitioners, we did not find longer REM sleep or SWS periods in meditation practitioners or differences in sleep efficiency or #awakenings, but meditators did show a tendency for a lower overall sleep time. This is consistent with the results of a study on Mindfulness-Based Cognitive Therapy, whereas meditation practitioners showed more cortical arousal and more awakenings during nighttime sleep than did controls (Britton et al., 2010). On the other hand, in another study, experienced long-term Buddhist practitioners (Ferrarelli et al., 2013) had a lower total sleep time, like the trend in our study, but also more awakenings.

The fact that we found no associations between meditation experience and sleep latencies, sleep efficiency or duration/proportion of individual sleep stages may be due to the fact that our sample contained no expert meditation practitioners, typically quantified as having more than 10 000 h of meditation practice (Brefczynski-Lewis et al., 2007). Because all our participants are considered meditation novices or at best intermediate practitioners, it is possible that the short daytime naps taken were insufficiently sensitive to reveal subtle group differences. Future studies might require all-night recordings or protocols that unmask subtle group differences by augmenting homeostatic pressure or REM sleep propensity.

#### Sleep Spindles

We found sleep spindle density differences between meditators and controls, with meditators unexpectedly showing reduced sleep spindle density in occipital derivations. Reduced spindle density has been suggested as a marker of neurodegeneration (Ktonas et al., 2007; Latreille et al., 2015) and psychopathological conditions such as schizophrenia (Wamsley et al., 2012; Schilling et al., 2016). Our finding is also unexpected because practice of meditation has been associated with increased neuroplasticity (Lazar et al., 2005; Slagter et al., 2011; Kang et al., 2012), and is thought to have neuroprotective effects against cognitive decline (Gard et al., 2014) and psychopathology (Shonin et al., 2015). For instance, in one study (Pattanashetty et al., 2009) Vipassana practitioners showed no age-related decline in slow-wave sleep and REM sleep compared to controls. Our results are interpreted with caution since these exploratory analyses would not withstand error correction for multiple comparisons (e.g., the Bonferroni correction). Future work should assess sleep spindles in meditation practitioners over a whole night of sleep and with a larger sample size.

Sleep spindles have also been suggested to play a role in maintaining sleep in face of environmental stressors, especially noise (Cote et al., 2000; Dang-Vu et al., 2010, 2011; Lecci et al., 2017). Reduced sleep spindles, in addition to other markers of physiological arousal during sleep in meditation practitioners, may represent a general developed trait of increased alertness/awareness of the environment during sleep. While in normal populations sleep fragmentation may represent a pathological hyper-vigilance that may lead to insomnia and increased stress levels (Mezick et al., 2009; Dang-Vu et al., 2015) in meditation practitioners, it may not have the same negative effect due to practices that emphasize non-reactive awareness, an active monitoring of the contents of awareness, and a general increase in alertness (MacLean et al., 2010). These effects, however, tend to depend on proficiency in meditation practice: meditators in early stages of practice report more fatigue and sleepiness, while more experienced practitioners report greater alertness (Britton et al., 2014).

Brain synchronization mechanisms have been proposed to be a marker of neuroplasticity, reflecting processes of strengthening of neuronal connections and creation of new, experience-dependent, networks (Engel et al., 2001). These processes were shown to have a strong top-down attentional component (Steinmetz et al., 2000). Long-term meditation practitioners generate long-distance phase-synchrony during a compassion meditation practice, and express EEG signatures different from those of controls during both active practice and the resting state (Lutz et al., 2004). This long-distance synchrony has been proposed to be a marker of cognitive integration (Lachaux et al., 1999; Varela et al., 2001), and alterations in neural synchrony patterns can be seen as markers of psychopathology (Uhlhaas and Singer, 2006). Sleep spindles involve synchronization on the level of thalamo-cortical communication in NREM sleep (Steriade and Timofeev, 2003). Recently, slow spindles were reported to be associated with brain synchrony over longer circuits, and faster spindles with more local connectivity (Zerouali et al., 2014). Thus, involvement of slow sleep spindles in procedural memory consolidation in meditation practitioners may reflect large-scale reorganization of brain connectivity – perhaps an increased efficiency of these networks – which may positively affect many cognitive skills, including memory.

Meditation practitioners, therefore, may develop a trait-like neurophysiological profile that involves both daytime and sleep changes. This trait may be similar to that of individuals with high dream recall: in a recent study we showed that increased fast but decreased slow sleep spindles correlate with dream recall frequency (Nielsen et al., 2016). The latter is a measure previously linked with higher reactivity to auditory stimuli both in wake and during sleep (Eichenlaub et al., 2014a), and with increased activity in the temporoparietal junction and medial prefrontal cortex – areas typically associated with attention and memory (Eichenlaub et al., 2014b). This finding is consistent with observations in our current study: all our participants had high dream recall, and meditators had fewer slow sleep spindles than did non-meditating controls, possibly reflecting their increased alertness in sleep.

Finally, it is possible that meditation subsumes some of the beneficial functions of sleep. While meditation practice is generally associated with increased self-reported well-being, health and life quality, during intensive meditation practice, e.g., a meditation retreat, meditators require less sleep (Kornfield, 1979), and exhibit physiological arousal signs, such as increased gamma coherence during slow wave sleep (Ferrarelli et al., 2013). And while sleep plays an important role in memory consolidation, restful wakefulness (as opposed to active wakefulness) also promotes learning (Brokaw et al., 2016). It is possible, therefore, that daytime meditation practice is sleep-like in nature and thus reduces sleep pressure and alter subsequent sleep architecture.

### Limitations

Due to the exploratory nature of this study, these results need to be interpreted with caution. The study design has three important limitations. First, we did not have a waking control group to test whether the Wii Fit task has a sleep-dependent component. The WiiFit task was selected because, unlike most traditionally used procedural tasks (e.g., finger tapping or rotor pursuit), it involves the whole body as opposed to just hand-eye coordination. Despite the lack of control group, our results indicate that in non-meditators, the task was associated with REM sleep duration – a finding well in line with much research on sleep-dependent consolidation of procedural memory. Second, some of the sleep differences between meditators and controls could be attributed to the fact that our meditators spent 10 min in meditation before bedtime, and that we did not have a group of meditators who did not meditate or controls who meditated. Vipassana meditators are encouraged to practice for 1 h every day. Thus, a 10-min session before bedtime was chosen to make sure that all meditators practiced before bedtime in the laboratory, ensuring that everyone in that group has some amount of practice on that day (including individuals who prefer to meditate in the evening/during the day). In addition, even if sleep differences could be influenced by meditation just before bedtime, group differences in the relationship between sleep architecture and learning in meditators and controls cannot be explained just by a very short meditation practice prior to bedtime.

Lastly, many of our comparisons (group comparisons for spindle density and correlations between task performance and sleep characteristics) would not survive conservative error correction, such as Bonferroni correction. In addition, the observed relationship between fast occipital spindles and task improvement in meditators was a trend (*p* < 0.1). Our project is an exploratory study, first of its kind, comparing sleep-dependent procedural learning in meditators and controls. The comparisons used (both multiple *t*-tests and correlations) were inherent to the study design and were planned in advance. It has been suggested that applying conservative error corrections in exploratory studies with planned comparisons may erase theoretically relevant effects (Perneger, 1998; Armstrong, 2014). Further, recent debate regarding an overreliance on *p* values (see recent issue of *The American Statistician*, Wasserstein et al., 2019), brought to question the wisdom of current practices that typically use *p* values for making dichotomous decisions regarding group differences. It has been proposed that effect size or confidence interval measures should supplement *p* values in making a decision on whether or not study results warrant further attention. In our study, the selective significant group differences in occipital spindles had a small to medium effect size, and magnitude of correlations between occipital spindle density and performance on the task was also moderate. Further research is needed to characterize sleep microarchitecture of meditation practitioners, and to elucidate potential changes in neurobiological substrates of motor learning associated with meditation practice.

## Conclusion

The relationship between improvement on a full-body procedural task and sleep architecture was distinctly different between meditators and controls. In meditation practitioners, task improvement was correlated with occipital spindle density in N2 sleep but not with the duration of any sleep stages; whereas in controls, task improvement was associated with increased time in REM sleep, but not with N2 sleep spindles. Both patterns of sleep changes have been observed in prior research although never linked differentially to meditation experience as in the present study.

Our finding of different patterns of sleep-dependent memory consolidation for meditating and non-meditating controls can be accounted for by at least two explanations. On the one hand, the body-focused practices of meditation may contribute to large-scale changes in learning, including changes in sleep microarchitecture and sleep-dependent memory consolidation. On the other hand, similarities between meditation practice and habitual napping may confer additional sleep-related or sleep-like advantages to meditators.

## Data Availability Statement

The raw data supporting the conclusions of this article will be made available by the authors, without undue reservation, to any qualified researcher.

## Ethics Statement

The studies involving human participants were reviewed and approved by Sacre-Coeur Hospital IRB. The patients/participants provided their written informed consent to participate in this study.

## Author Contributions

ES and TN led the design of the study, data collection, analysis, and interpretation. ES was the lead writer of the manuscript. SD, AS-R, CB-C, and MC collected data. ES, SD, AS-R, and TP conducted spindle and task analyses. DS contributed to editing, manuscript writing, and revision.

## Conflict of Interest

The authors declare that the research was conducted in the absence of any commercial or financial relationships that could be construed as a potential conflict of interest.
